# Green tea consumption increases sperm concentration and viability in male rats and is safe for reproductive, liver and kidney health

**DOI:** 10.1038/s41598-020-72319-6

**Published:** 2020-09-17

**Authors:** Chinyerum Opuwari, Thomas Monsees

**Affiliations:** 1 Department of Pre-Clinical Sciences, University of Limpopo, Polokwane, South Africa; 2 Department of Medical Biosciences, University of the Western Cape, Cape Town, South Africa

**Keywords:** Cell biology, Physiology, Anatomy

## Abstract

Green tea is a popularly consumed beverage worldwide and contains polyphenols, whose antioxidant activities could improve sperm parameters and fertility thereof. We investigated the effect of green tea on the male rat reproductive system as well as its safety. Male Wistar rats were administered 2 and 5% aqueous extract of green tea for 52 days’ ad libitum, while the control group received tap water. Total polyphenol, flavanol, flavonol and soluble solids significantly increased in a concentration-dependent manner in vitro (*P* < 0.01). Weights of body, testis, epididymis, prostate gland, seminal vesicles, and liver, serum levels of testosterone, ferric reducing antioxidant power, creatinine, and sperm motility, remained unchanged (*P* > 0.05). Kidney weight, sperm concentration and vitality, spontaneous acrosome reaction increased (*P* < 0.05), while alanine transaminase and aspartate transaminase levels decreased (*P* < 0.05). Catalase, superoxide dismutase, glutathione and lipid peroxidation remained unchanged in the testes, liver and kidney (*P* > 0.05). Histological sections of testis, epididymis, kidney and liver showed no conspicuous alteration. Diameter and epithelial height of seminiferous tubule decreased, while caudal epididymis epithelial height increased (*P* < 0.01). Consumption of green tea in the conditions used in the present study seems to be safe and improved sperm parameters. However, subtle structural changes observed in the decreased diameter and epithelial height of the seminiferous tubule and increased acrosome reaction needs further investigation.

## Introduction

*Camellia sinensis* (L.) Kuntze (Fam. Theaceae) consists of three types based on its level of fermentation (oxidation) as unfermented (white and green teas), partially fermented (oolong tea) and completely fermented (black tea)^1^. White tea is obtained by an instant steaming of young tea leaves or buds, followed by drying. In contrast, green tea is obtained by immediately steaming freshly harvested mature tea leaves, resulting in the minimal oxidation of the naturally occurring polyphenols in its leaves^1,2^. Oolong tea is produced by shorter fermentation process while black tea is obtained by rolling of the leaves, followed by a period of 90–120 min’ oxidation^3,4^.

Green tea is a popularly consumed beverage worldwide, especially in China, Japan and other Asian countries, including India^5^. It accounts for 20% of tea consumption in the world^6^, with black tea being the most consumed and white tea, the least^7^. The leaves of green tea contain 30–40% polyphenols (which contains 80% flavonoids), fibres (26%), proteins (15%), lipids (2–7%), vitamins and minerals (5%), methylxanthines (3–4%) and pigments (1–2%)^2,8^. Green tea composition may vary and depends on its geographical location, agricultural practices, as well as the properties of the plant itself^8^. Green tea consumption could be beneficial for risk reduction in cardiovascular diseases, obesity, diabetes, neurodegenerative diseases and cancer as well as weight loss^9,10^. Although the beneficial effect of consumption of green tea on the overall risk of cancer remains unproven as findings from epidemiological studies revealed inconsistent results^11^. The health benefits of green tea are attributed mostly to the polyphenolic compounds especially the catechins (e.g. epicatechin (EC), epigallocatechin (EGC), epicatechin-3-gallate (ECG) and epigallocatechin-3-gallate (EGCG)), with EGCG being the most abundant and effective^10,12^. Unfermented teas have high polyphenolic content, with catechin derivatives, EGCG being the most abundant and powerful antioxidant^13^. Although, a higher amount of antioxidants, as well as antioxidant potential, are seen in white tea compared to green tea in a dose-dependent manner^1,14^.

Intake of three to four cups of green tea (equivalent to 2 g) three times a day is to be safe for administration in a long term basis^15^. Human intervention and bioavailability studies involving low to moderate doses of green tea preparations or EGCG reported no adverse effect^16–18^. However, other studies revealed that administration of high doses of green tea or EGCG resulted in liver and renal toxicity^12,15,19,20^, in which its polyphenol catechins could be the causative agent^20^. High levels of serum aspartate transaminase (AST), alanine transaminase (ALT), and creatinine, respectively indicate liver and renal toxicity or injury as ALT and AST are biomarkers of the liver structure and function, while creatinine is a reliable renal biomarker^21,22^. The harmful effects of green tea have also been attributed to the pro-oxidative property when consumed at high doses^23^. Repeated administration of green tea polyphenols (GTPs) (750 mg/kg) caused a hepatic injury^24^. Oral administration of EGCG (1,500 mg/kg) increased ALT in plasma and reduced survival rate in CF-1 mice^9^. Mazzanti et al. also showed a link between the intake of green tea supplement and liver damage^25^. Salminen et al. demonstrated that green tea extract (500 and 1,000 mg/kg) potentiated acetaminophen-induced hepatotoxicity in mice^26^. Low doses of GTPs (0.01 and 0.1%) but not the high dose of GTPs (1%) ameliorated dextran sulfate sodium (DSS) increased ALT and AST levels in mice^22^. Administration of high dose GTPs (1%) to male mice caused renal toxicity, as marked by increased serum creatinine level, and increased thiobarbituric acid-reactive substances (TBARS) in both kidney and liver^22^. Protective effects of green tea extract were also demonstrated against nephrotoxic agents in rats^27–29^.

The testis performs two crucial functions, that is, spermatogenesis (production of spermatozoa) and steroidogenesis (production of steroid hormones), which are regulated by gonadotropins and locally synthesised factors^30^. The whole spermatogenic cycle takes 52 days in rats^31^. Previous studies have indicated inconsistent results on the exposure of green tea to the male reproductive system. Yassa et al. showed that administration of the aqueous green extract (70 mg/kg/day) for 90 days did not affect the body weight, serum testosterone, and the histological appearance of the seminiferous tubules of the testes was normal^32^. Oral administration of aqueous extract of green tea (70 mg/kg/day) to rats for 63 days did not affect weights of the body and reproductive organs, sperm concentration and viability and diameter of the seminiferous tubule^33^. Oral administration of 1.25% and 5% green tea extract (polyphenone-60) mixed with powdered CE-2 diet to male rats for 2, 4 and 8 weeks’ ad libitum decreased weight of the body, testes, and prostate gland, but increased levels of luteinising hormone (LH) and testosterone, with no marked difference in the histological sections of the testis at the highest dose^34^. Consumption of 2.5% and 5% green tea extract (1 ml/100 g body weight) for 26 days resulted in impaired spermatogenesis through the loss of matured and elongated spermatids. Decreased net weight gain and testis weight, sperm count and motility, Δ5 3β- and 17 β-hydroxysteroid dehydrogenase and testosterone levels, with increased follicle-stimulating hormone (FSH) and LH in adult male rats, was also observed^35,36^.

Furthermore, intraperitoneal injection of EGCG (85 mg/kg body weight) for 2–7 days caused acute body weight loss, and reduction in weight of prostate gland and testis as well as the level of testosterone and LH^37^. Administration of lower concentrations of EGCG (2 µM and 20 µM) to human sperm enhanced sperm quality through the increased motility, viability and capacitation. In comparison, administration of a high concentration of EGCG (60 µM) caused a deleterious effect^38^. Supplementation of sperm storage media with tea extract increased sperm viability in a dose-dependent manner^1^. Other studies have shown the ameliorative or cytoprotective property of green tea following the administration of testicular toxicants such as cadmium and acrylamide or by the induction of oxidative stress in animal models^32,33,39–41^. In this study, we report on the effect of green tea in the male rat reproductive system after a complete spermatogenic cycle of 52 days as well as its safety by considering the liver and kidney functions.

## Results

### Chemical analysis of green tea

A significant increase in total polyphenol (*P* < 0.0001), flavanol content (*P* < 0.01) and flavonol content (*P* < 0.0001) were observed in the 5% green tea extract compared to that of 2%. The soluble solid content (*P* < 0.0001) as well as the ferric reducing power (*P* < 0.0001) was also higher in the 5% in green tea extract compared to 2% green tea extract) (Table 1).

**Table 1:** Chemical analysis of an aqueous extract of green tea.

Chemical analysis (unit)	2% Green tea	5% Green tea	*P*-value
Total polyphenol (mg Gallic acid equivalent/ml)	3.49 ± 0.22	8.66 ± 0.44	< 0.0001
Flavanol (mg catechin/ml)	1.43 ± 0.14	4.17 ± 0.51	0.0020
Flavonol (mg quercetin equivalent/ml)	0.06 ± 0.01	0.13 ± 0.01	< 0.0001
FRAP (mM)	8.25 ± 0.30	18.48 ± 1.27	< 0.0001
Soluble solids (mg/ml)	4.89 ± 0.35	11.81 ± 0.66	< 0.0001

Values are the means ± SEM of 12 replicates.

*FRAP* ferric reducing antioxidant power.

### Fluid intake and weight gain

**Figure 1:**
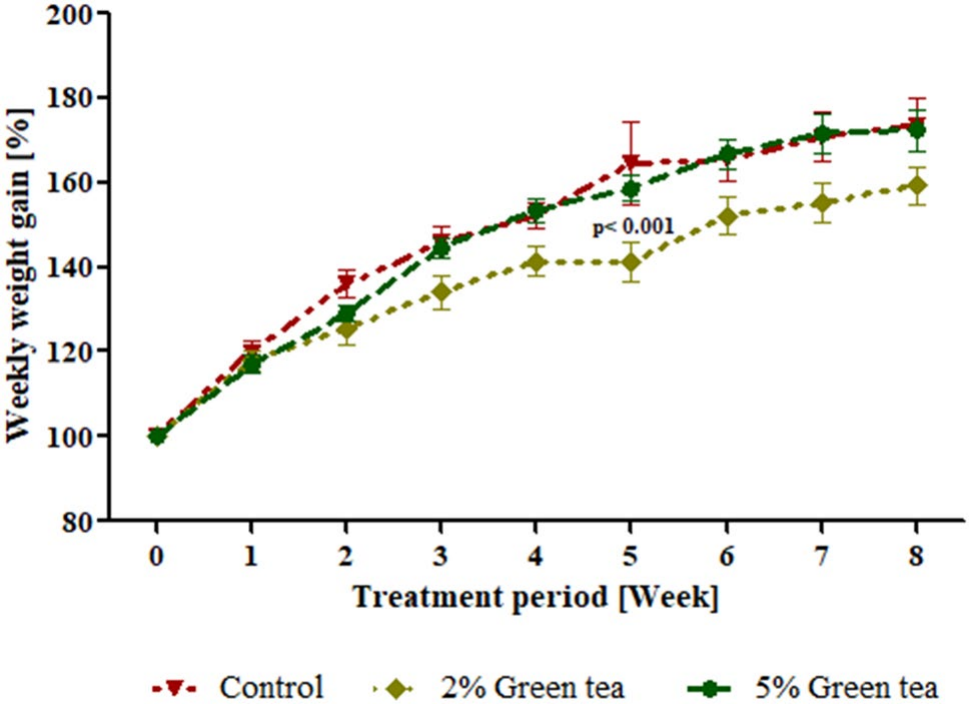
Weekly weight gain for the period of treatment in male rats. Values represented are the mean ± SEM of 6 animals per group. ***p < 0.0001 compared to the control.

Overall, fluid intake in male rats exposed to green tea (2% or 5%) did not differ to the control group (Table 2; *P* > 0.05). Bodyweight gain of animals in all groups increased progressively throughout treatment (Fig. 1). Rats treated with green tea showed no change in the weekly weight gain compared to the control (*P* > 0.05), although the 2% green tea treated group showed a reduced weight gain at week five compared to the control (Fig. 1; *P* < 0.0001). The total weight gain of the rats remained unchanged after consumption of green tea for 52 days (Table 2; *P* > 0.05). Furthermore, the net body weight gain of the control 2% and 5% green tea treated groups were 72.5%, 59.1% and 72.3% respectively (data not shown). Besides, 2% and 5% green tea caused no change in the weight of the testis, epididymis, prostate, seminal vesicles and liver on the male rats (Table 2; *P* > 0.05), while a substantial rise in the weight of the kidney was observed following exposure to 5% green tea (Table 2; *P* < 0.05).

**Table 2:** Effect of consumption of aqueous extract of green tea for 52 days on fluid intake, body and organ weights of male Wistar rats.

	Control	2% Green tea	5% Green tea
Average daily fluid intake (ml/100 g BW)	13.43 ± 0.58	12.88 ± 0.47	12.41 ± 0.60
Total weight gain (g)	151.80 ± 10.22	141.70 ± 9.57	158.80 ± 10.06
Testis (g)^a^	0.47 ± 0.02	0.48 ± 0.04	0.46 ± 0.04
Epididymis (g)^a^	0.18 ± 0.01	0.17 ± 0.01	0.14 ± 0.04
Seminal vesicles (g)^a^	0.41 ± 0.01	0.39 ± 0.05	0.38 ± 0.03
Prostate gland (g)^a^	0.13 ± 0.01	0.13 ± 0.03	0.13 ± 0.04
Kidneys (g)^a^	0.85 ± 0.02	0.87 ± 0.05	0.93 ± 0.05*
Liver (g)^a^	4.57 ± 0.14	4.48 ± 0.37	4.62 ± 0.25

Values represents mean ± SEM of six male rats per group.

*BW* body weight.

*P < 0.05 compared to the control.

^a^Relative organ weight = organ weight/final body weight × 100.

### Serum analysis

Green tea (2% and 5%) did not affect the level of testosterone produced in the male rats (Table 3; *P* > 0.05). However, a positive trend in the level of testosterone was observed in a dose-dependent manner (ANOVA-trend analysis, repeated measure: *P* = 0.029). Ferric reducing antioxidant power in serum remained unchanged following the 52 day’s treatment (Table 3; *P* > 0.05). Creatinine remained unchanged in its serum level in the treated rats (Table 3; *P* > 0.05). The AST level in serum, as shown in Table 3 dropped significantly in rat exposed to 2% and 5% green tea (*P* < 0.05). Repeated measure ANOVA trend analysis showed an increase in the level of AST in serum (*P* = 0.0050) at the higher tea concentration used. Serum ALT activity decreased significantly in the treated groups (Table 3; *P* > 0.05). Although not significant, a trend to a decreased level of serum ALT activity was observed with increasing concentrations of green tea (Repeated measure ANOVA-trend analysis: *P* = 0.1430).

**Table 3:** Analysis of serum following treatment of male Wistar rats with green tea.

	Testosterone (ng/ml)	FRAP (µM)	Creatinine (mg/l)	AST (UI/l)	ALT (UI/l)
Control	5.3 ± 0.8	328.1 ± 26.0	35.4 ± 4.4	46.0 ± 3.6	47.1 ± 3.2
2% Green tea	6.0 ± 1.4	329.0 ± 35.0	44.6 ± 3.3	22.0 ± 2.3***	35.5 ± 1.1*
5% Green tea	8.7 ± 0.7	288.0 ± 52.8	46.4 ± 6.3	26.0 ± 1.1***	31.3 ± 1.3**

Values represented are the mean ± SEM of 6 animals per group.

*FRAP* ferric reducing antioxidant power, *AST* aspartate transaminase, *ALT* alanine transaminase.

*P < 0.05; **P < 0.01, ***P < 0.0001 compared to control.

### Sperm parameter analysis

**Figure 2:**
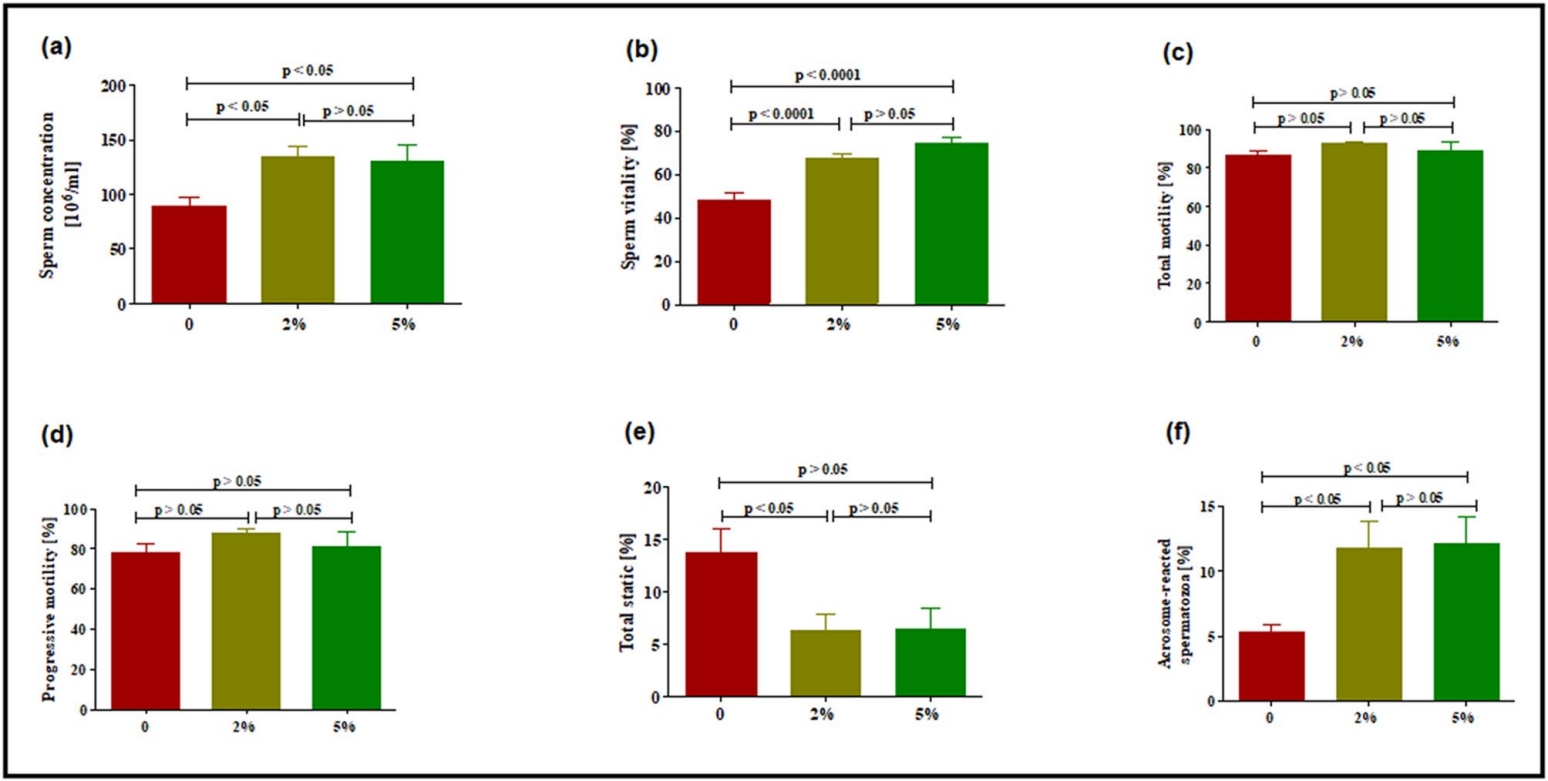
Effect of green tea on sperm parameters. (**a**) Sperm concentration, (**b**) sperm vitality, (**c**) total motility, (**d**) progressive motility, (**e**) total static sperm, (**f**) acrosome reacted spermatozoa. Values are represented as mean ± SEM after 52 days’ treatment. The number of rat per group = 6; at least 200 spermatozoa per animal were analysed.

Green tea significantly increased sperm concentration (*P* < 0.05) (Fig. 2a) and sperm vitality control (*P* < 0.05). The increase in sperm vitality was up to 40% and 54.8% in the 2% and 5% green tea treated groups, respectively (Fig. 2b). Total motility (Fig. 2c) and total progressive motility (Fig. 2d) remained unchanged (*P* > 0.05) in both treated groups. Also, green tea caused a significant decline in the percentage of total static spermatozoa (*P* < 0.05; Fig. 2e). Further to these, sperm velocity parameters, that is, ALH, BCF, LIN, STR, VAP, VCL, VSL, and WOB remained unchanged (*P* > 0.05; Table 4). following exposure of male rats to 2% and 5% green tea. Lastly, the percentage of acrosome reacted spermatozoa significantly increased (*P* < 0.05; Fig. 2f).

**Table 4:** Effect of consumption of aqueous extract of green tea for 52 days on sperm velocity parameters of male Wistar rats.

	Control	2% Green tea	5% Green tea
VSL (µm/s)	51.7 ± 8.6	59.6 ± 3.0	61.4 ± 10.7
VCL (µm/s)	274.0 ± 18.9	297.5 ± 20.9	274.9 ± 37.7
VAP (µm/s)	99.2 ± 8.9	113.0 ± 7.0	105.7 ± 12.7
ALH (µm)	10.3 ± 0.4	10.8 ± 0.3	10.1 ± 0.8
LIN (%)	18.5 ± 1.9	20.2 ± 0.6	22.1 ± 1.8
STR (%)	51.9 ± 4.0	53.0 ± 1.6	57.1 ± 4.9
WOB (%)	36.1 ± 1.0	38.1 ± 0.5	38.9 ± 0.8
BCF (Hz)	6.4 ± 0.6	6.1 ± 0.3	7.3 ± 1.1

Values represented are the means ± SEM of 6 animals per group, and 200 sperm per animal were analysed.

*VSL* straight-line velocity, *VCL* curvilinear velocity, *VAP* average path velocity, *ALH* the amplitude of lateral head displacement, *LIN* linearity, *STR* straightness, *WOB* wobble, *BCF* beat cross frequency.

### Histology and morphometric measurements

**Figure 3:**
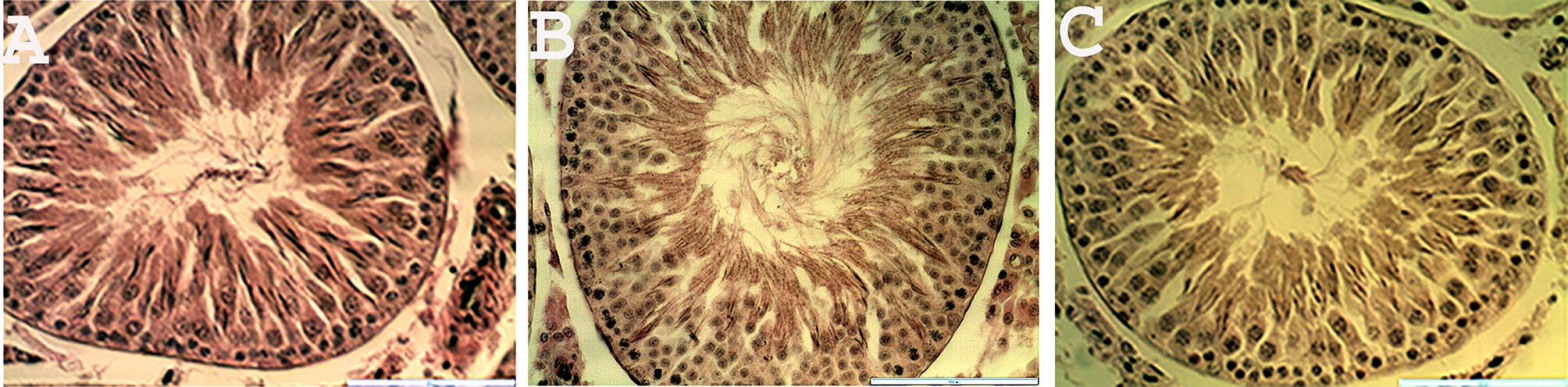
Effect of consumption of aqueous extract of green tea for 52 days on the testes of male Wistar rats. (**A**) Control; (**B**) 2% Green tea; (**C**) 5% Green tea. Bar: 100 µm.

**Figure 4:**
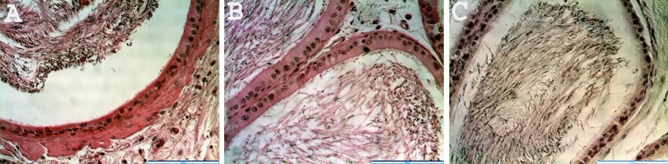
Effect of consumption of aqueous extract of green tea for 52 days on the cauda epididymis of male Wistar rats. (**A**) Control; (**B**) 2% Green tea; (**C**) 5% Green tea. Bar: 100 µm.

**Figure 5:**
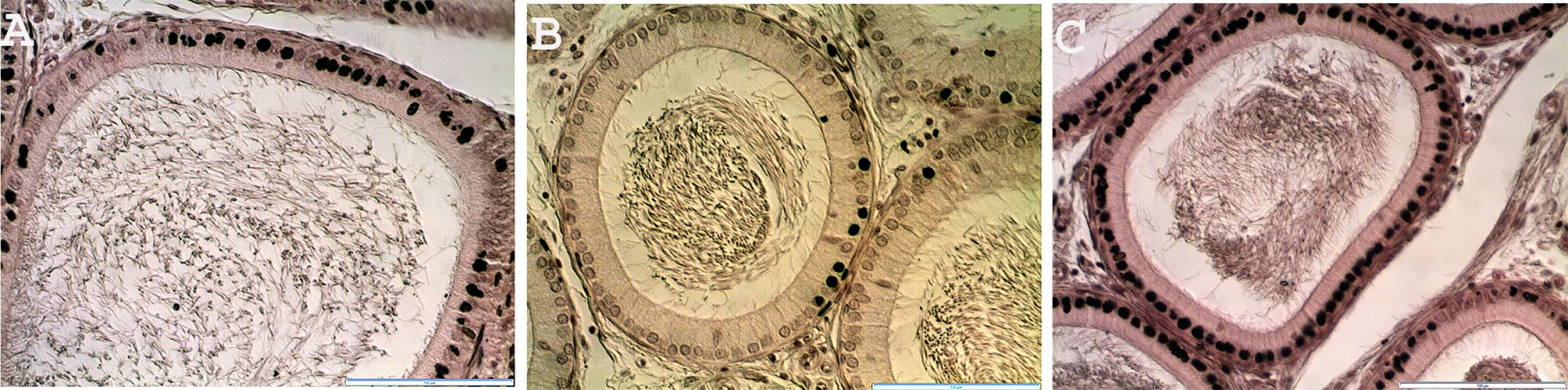
Effect of consumption of aqueous extract of green tea for 52 days on the caput epididymis of male Wistar rats. (**A**) Control; (**B**) 2% Green tea; (**C**) 5% Green tea. Bar: 100 µm.

**Figure 6:**
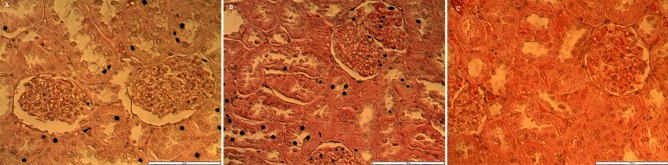
Effect of consumption of aqueous extract of green tea for 52 days on the kidney of male Wistar rats. (**A**) Control; (**B**) 2% Green tea; (**C**) 5% Green tea. Bar: 100 µm.

**Figure 7:**
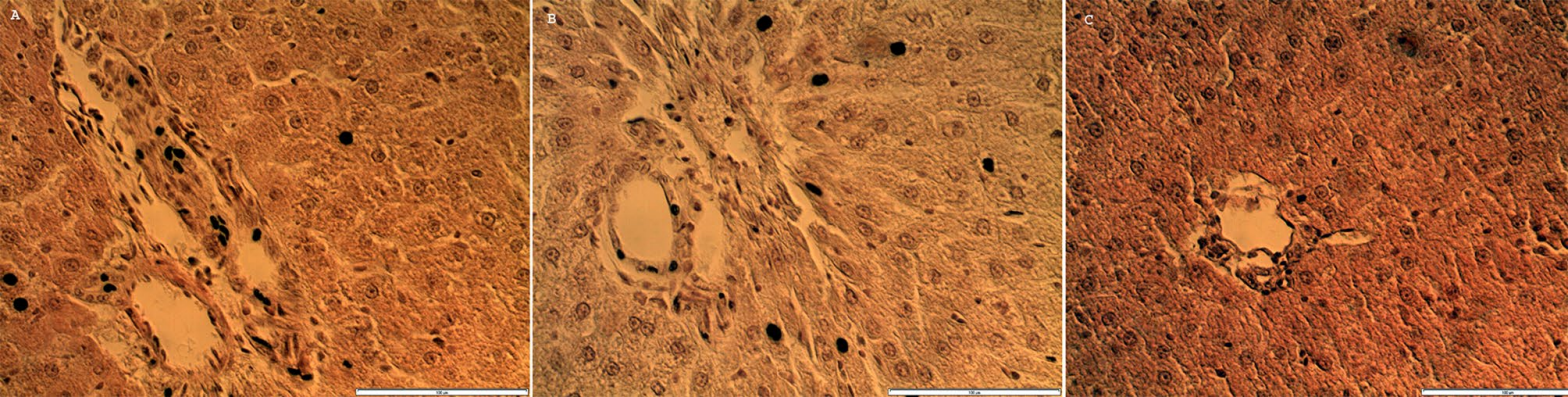
Effect of consumption of aqueous extract of green tea for 52 days on the liver of male Wistar rats. (**A**) Control; (**B**) 2% Green tea; (**C**) 5% Green tea. Bar: 100 µm.

The histological observation of the testes in the treated rats showed no noticeable distortion in the architecture of the cells compared to the control (Fig. 3). All stages of spermatogenesis were present, and copious spermatozoa in the lumen of the seminiferous tubule in the treated groups, like the control. Furthermore, sections of the epididymis (cauda and caput) also showed no difference in structure compared to the control group (Figs. 4 and 5). On the contrary, the diameter (2% and 5%; *P* < 0.0001) and epithelial heights (2%, *P* < 0.05) of seminiferous tubules of the treated groups reduced significantly (Table 5). Green tea extract (2%) significantly increased the epithelial height of the cauda epididymis (p < 0.05); however, the epithelial height of the caput epididymis remained unchanged (p > 0.05) (Table 5). Furthermore, the histological sections of the treated kidney and liver depicted standard architecture compared to the control (Figs. 6 and 7).

**Table 5:** Effect of consumption of aqueous extract of green tea for 52 days on the measurements of the seminiferous tubules and epididymis.

	Seminiferous tubule		Epididymis epithelial height	
	Diameter (µm)	Epithelial height (µm)	Caput (µm)	Cauda (µm)
Control	281.4 ± 3.0	92.8 ± 1.4	24.1 ± 0.5	16.8 ± 0.5
2% Green tea	263. 8 ± 2.4***	88.4 ± 1.1*	26.0 ± 0.8	19.4 ± 0.8*
5% Green tea	262.8 ± 2.7***	92.0 ± 1.2	25.8 ± 0.6	17.9 ± 0.6

Values represents mean ± SEM of six male rats per group.

*P < 0.05; ***P < 0.0001.

### Antioxidant enzymes and lipid peroxidation in Tissues

Table 6 shows the results on lipid peroxidation and antioxidant enzymes in the testes, liver and kidney following treatment with green tea (2% and 5%). No change in the level of MDA in the testes, liver and kidney (*P* > 0.05; Table 6). Similarly, GSH, CAT and SOD levels in the kidney, liver and testes remained unchanged (*P* > 0.05).

**Table 6:** Effect of consumption of aqueous extract of green tea for 52 days on the antioxidant activities in the testis, kidney and liver of male Wistar rats.

Organ	Antioxidant assays (units)	Control	2% Green tea	5% Green tea
Testis	CAT (µmole/min/mgprot)	1.5 ± 0.2	1.6 ± 0.1	1.3 ± 0.1
	GSH (mM/mgprot)	0.1 ± 0.0	0.1 ± 0.0	0.1 ± 0.0
	SOD (U/mgprot)	0.4 ± 0.0	0.4 ± 0.0	0.4 ± 0.0
	MDA (nmole^−1^/mgprot)	0.5 ± 0.1	0.4 ± 0.0	0.5 ± 0.1
Kidney	CAT (µmole/min/mgprot)	1.8 ± 0.1	2.1 ± 0.2	1.6 ± 0.1
	GSH (mM/mgprot)	0.1 ± 0.0	0.1 ± 0.0	0.1 ± 0.0
	SOD (U/mgprot)	1.2 ± 0.1	1.0 ± 0.1	1.2 ± 0.2
	MDA (nmole^−1^/mgprot)	2.9 ± 0.3	3.1 ± 0.4	2.9 ± 0.6
Liver	CAT (µmole/min/mgprot)	2.3 ± 0.2	1.9 ± 0.2	2.4 ± 0.2
	GSH (mM/mgprot)	0.1 ± 0.0	0.1 ± 0.0	0.1 ± 0.0
	SOD (U/mgprot)	1.0 ± 0.1	1.6 ± 0.3	1.2 ± 0.2
	MDA (nmole^−1^/mgprot)	3.3 ± 0.4	4.2 ± 0.8	2.9 ± 0.6

Values represented are the mean ± SEM of 6 animals per group.

*CAT* catalase, *GSH* glutathione, *MDA* malondialdehyde, *SOD* superoxide dismutase.

## Discussion

The results on the effect of green tea extract or its polyphenols (such as EGCG) on the male reproductive functions and spermatogenesis are conflicting^32–35,37^. Also, a high dose of green tea extract caused renal toxicity and hepatotoxic effects^12,15,19^. Hence, we aim to report on the overall effect of consumption of green tea ad libitum on the male reproductive system after the completion of one spermatogenic cycle in rats, as well as on the kidney and liver functions.

Green tea leaves contain 30–40% polyphenols^2,8^. Similar to previous studies^1,14^, in vitro analysis of green tea in our study revealed the presence of these polyphenols and further demonstrated a concentration-dependent increase in total polyphenols, flavanol, flavonol, and soluble solids. Further analysis of the antioxidant capacity of green tea in vitro, using FRAP showed a concentration-dependent increase; however, it remained unchanged in the serum of the treated male rats. These were indicating a possible higher antioxidant property with higher concentrations of the extract. Our study however, also showed no change in the serum FRAP after drinking green tea in vivo, which supports the findings of Maxwell & Thorpe^42^. Contrary to our study, consumption of green tea significantly increased plasma FRAP in humans^43^. The possible explanation could be due to the differences in bioavailability of green tea, as bioavailability and transformation of EGCG, the predominant catechin in green tea, differs significantly between humans, mice and rats^44^. Low bioavailability of EGCG was also demonstrated following administration of GTP as the sole source of drinking fluid in rats, with a higher level seen in mice^45^.

The animals consumed green tea extract (2 and 5%) ad libitum*,* and their average intake was comparable to the control group and corresponds to approximately 40 cups per day in an 80 kg man. The study demonstrated a progressive rise in the weekly weight of animals, with no obvious signs of clinical toxicity or behavioural changes in the rats, indicating the well-being of the animals during the treatment period. Furthermore, body weight gain of the treated rats also remained unchanged. Similar, to our study, administration of green tea (70 mg/kg/day) to rats for 63 days or 90 days did not affect body weight gain^32,33^. However, a transient reduction in body weight was observed at week 5 in the 5% treatment group and may be attributed to taste aversion and reduced food intake. Kao et al. however, reported that EGCG (a predominant green tea polyphenol) reduced body weight in rats^37^. Green tea catechins (especially EGCG) are responsible for the reduced body weight by decreasing the differentiation and proliferation of adipocytes during lipogenesis^46^. Also, a significant rise in weight loss was observed in humans following consumption of a high dose of green tea for 12 weeks. It may be attributed to inhibition of ghrelin secretion, which leads to increased adiponectin levels^47^.

Although our study, however, showed a remarkable rise in kidney weight, the creatinine level remained unchanged, suggesting that green tea did not damage renal function. Another study revealed that high concentrations of green tea catechins (5%) increased the kidney weight, with no histopathologcal observation^48^. Also, l-theanine (a predominant free amino acid found in tea) was shown to increase kidney weight in rats, with no histological correlations^49^. Higher protein or amino acid intake modulates renal hemodynamic by increasing renal blood flow and elevating intraglomerular pressure that results in an increased glomerular filtration rate (GFR) as as well an increased kidney volume and weight^50^. The increased weight in our study may be attributed to the catechins or amino acid (l-theanine) content in green tea and was considered not to be toxicologically significant as there was no obvious histopathological changes in the kidney together with a comparable level of creatinine as the control. Besides, the weight of the liver remained unchanged, with significantly reduced levels of AST and ALT. Both of these findings suggest that consumption of green tea at least in rats had no detrimental effects, as also supported by the histological sections of the treated kidneys and liver. Our study is in corroboration with Bun et al.^6^, as no sign of evidence of distinctive hepatotoxicity was found in green tea treated rats, as this study showed no hepatic functional disorders based on the levels of ALT and AST in serum, as well as histological sections of the liver. The results of the current study are in line with the literature data suggesting hepatoprotective properties of green tea preparations^51–53^. Takami et al. revealed that prolonged intake (90 days) of high concentrations of green tea catechins (5%) brought about hepatotoxic effects in male rats by the increased levels of ALT and AST^48^, which could suggest that the use isolated or pure polyphenols could be detrimental to the functioning of these organs. Furthermore, case-reports have associated the use of concentrated green tea-based supplements with liver toxicity in humans^54,55^.

Our study demonstrated that the level of testosterone (which plays a crucial role in spermatogenesis) remained unchanged, even though, a trend to increased values was observed with increasing concentration of green tea. Other studies showed that green tea extract and EGCG produced a substantial drop in testosterone level in Leydig cells in vitro^5,56^, while testosterone production in vivo studies showed a reduction^35,37^, increment^34^ or remained unchanged^32^ in male rats. A possible explanation for these variations could be due to the use of different forms of the plant (crude extract, or isolated compounds), modes of administration or length of treatment.

Reproductive organ weights are used as indicators of reproductive toxicity^57^, and a reduction of testicular weight is a sensitive parameter for interpretation of male gonadal toxicity^58^. In line with the observed level of testosterone and similar to a previous study^33^, intake of green tea in this study did not alter the weight of testosterone dependent organs (testis, epididymis, seminal vesicles and prostate gland). Also, histological sections of the testis and epididymis revealed no obvious alteration in their structures. Furthermore, sperm quality is also used as an important indicator of chemical-induced toxicity on testis^59^. While a study demonstrated that green tea extract did not affect sperm concentration and viability^33^, our present study showed that green tea significantly increased sperm concentration and sperm vitality, rather, sperm motility and velocity functions were unaffected. However, morphometric measurements in our study showed a significant reduction in the diameter and epithelial height of the seminiferous tubule. In contrast, epithelial heights of cauda epididymis increased, and that of the caput remained unchanged. Contrary to our study, green tea did not affect the diameter of the seminiferous tubule^33^; however, a reducing trend was observed. Reproductive toxicants are known to reduce the diameter and epithelial height of seminiferous tubule as well as impair spermatogenesis^59–61^. Our study however, demonstrated an increase in sperm viability, concentration, and epithelial heights of epidiydymis with no dinstinct distortion of the seminiferous tubules or loss of spermatogenic cells. The mechanism of action responsible for these increment is unclear and requires further investigation to understand the impact on the male reproductive functions and fertility outcome.

Bioactive compounds in tea such as catechins, caffeine, and l-theanine^62^ are shown to possess conflicting effects on the male reproductive system. For instance, low concentrations of caffeine (mainly derived from tea leaves) stimulates lactate (the preferred metabolic substrate for developing germ cells) production (5 and 50 µM), and increases expression of glucose transporters (50 µM; GLUTs) in human Sertoli cells (hSCs)^63^. In another study, administration of caffeine (200 mg/kg body weight) negatively affected the male reproductive functions as demonstrated by the reduced testicular and epidydimal weight, distortion of the histo-architecture of the seminiferous tubule and loss of spermatogenic cells, sperm count, motility and viability with increase abnormal sperm^64,65^. In addition, maternal consumption of caffeine during gestation (26 and 45 mg/kg) and lactation (25 and 35 mg/kg) was shown to also negatively affect male reproductive function in the male offspring rats as indicated by a significant reduction in testicular weight, diameter and epithelial heights of seminiferous tubules, testosterone production and sperm quality^66^. EGCG was shown to have an antiproliferative effect on hSCs (5 and 50 µM), although lactate production was maintained (5 and 50 µM) with an increased consumption of glucose and pyruvate (50 µM). However, it decreased the mitochondrial membrane potential (50 µM) and conversion of pyruvate to alanine (5 and 50 µM), which ensures the regular production of lactate^67^. l-Theanine, on the other hand, increased hSC proliferation, glucose consumption and mitochondrial membrane potential, although lactate production remained unchanged^68^. Also, supplementation of sperm storage media for up to 3 days either with caffeine (71 µg/ml), EGCG (82 µg/ml) or l-theanine (19 µg/ml) had no effect on sperm viability, while the supplementation with a combination of these three compounds significantly increased sperm viability, suggesting a synergestic effect of the components of tea rather than an individual bioactive compound^69^. Hence, a possible explanation for the increased sperm concentration and viability in our study might be attributed to an increased lactate production by the Sertoli cells or increased GLUT protein levels and activities, although further studies are required to validate this.

In order to fertilise an oocyte, capacitated spermatozoa must undergo acrosome reaction, and consists of the release of the hydrolytic enzyme in the secretory vesicle of the sperm acrosome, for the degradation of the zona pellucida of the oocyte. A premature or spontaneous acrosome reaction makes the spermatozoa incompetent to interact with the oocyte and thereby to impair its fertilisation rate^70^. This study revealed a significant increase in spontaneous acrosome reaction, posing a question on the ability of these spermatozoa to fertilise an oocyte. Production of F-actin during capacitation was shown to be crucial in the prevention of spontaneous acrosome reaction^71^. An increase in cyclic adenosine monophosphate (cAMP) results in the activation of protein kinase A (PKA) at the onset of capacitation, which indirectly controls the phosphorylation of protein tyrosine, hyperactivated motility and actin polymerisation^72^. Further to this, calmodulin kinase II (CAMKII) and phospholipase D (PLD) were shown to be major pathways responsible for the polymerisation of actin^71^. Tsirulnikov et al. further showed that PKA protects the sperm from undergoing spontaneous acrosome reaction by inducing actin polymerisation via PLD and CAMKII^73^. The possible explanation for the increased spontaneous acrosome reaction as seen in this study could be due to inhibition of PKA, actin polymerisation or CAMKII and PLD pathways, which needs to be further interrogated. Isotani et al. rather reported that acrosin-disrupted spermatozoa equally pierced the zona pellucida as the wild-type spermatozoa, rather, a delayed dispersal rate of cumulus oophorus cells caused a decrease in the number of fertilised oocyte and litter size^74^. Hence, the effect of green tea or its polyphenolic compounds on fertility rate must be further investigated to understand better the implication of spontaneous acrosome reaction observed in this study.

In comparison to the findings of this study, a previous study showed that black tea increased sperm vitality, motility, the epithelial height of the cauda epididymis, kidney weight, as well as spontaneous acrosome reaction while decreasing the activities of AST and ALT and diameter and epithelial height of the seminiferous tubule^75^, however, an increased creatinine activity was observed. Another study demonstrated the aphrodisiac property of black tea, accompanied by an increase in testosterone production in rats^76^. White tea, which is known to have a higher level of antioxidants compared to green, oolong and black teas^14^, stimulated lactate production in Sertoli cells, which provides nutritional support to the developing germ cells^77^, as well as increased rat sperm viability^1^. White tea was also shown to improve prediabetes-induced reproductive dysfunction through increment in glucose transporters 2 and 3 (GLUT 2 and GLUT 3) protein levels and phosphofructokinase 1 *(*PFK1) activity, sperm motility, with restoration of testicular lactate content and sperm viability^78^. In another study, white tea improved testiscular antioxidant potential and sperm concentration, and restored sperm quality (motility, morphology and viability) in prediabetic male rats^79^. These are suggestive of possible effects of the antioxidants in these plants on various aspects of male reproductive functions.

Most of the beneficial effects of green tea are attributed to the antioxidant and free-radical scavenging properties, its polyphenols and flavonols^80^. Our study showed that the levels of CAT, GSH, MDA, SOD were unaffected by green tea in the liver, kidney and testes of the treated rats, which indicates the safety of the plant and suggests that a balance between the scavenging activity of the antioxidant and generation of ROS is not compromised, thereby preventing oxidative stress under the tested conditions. Another study showed that testicular CAT and GSH remained unchanged while a significant increase in SOD was noted^33^. Green tea extract was shown to protect testicular tissue against oxidative damage by increasing its antioxidant defence mechanism as demonstrated by the increased SOD and GSH activities with a resultant decrease in lipid peroxidation^81^. Antioxidant therapy is used to eliminate, take up or reduce the formation of ROS. Antioxidant supplements are categorised based on their activities as a preventive antioxidant (e.g. lactoferrin), which prevents the formation of reactive oxygen species (ROS), and scavenging antioxidants (e.g. vitamins C and E), which removes already present ROS^82^. The antioxidants are also classified as enzymatic (natural) oxidants (includes glutathione reductase, catalase, superoxide dismutase) and non-enzymatic oxidants, (includes vitamins B, C and E, carotenoid, carnitine, cysteine), which are derived from fruits and vegetables^83^. For instance, intake of a low amount of vitamin C was associated with increased oxidative stress^84^, while high levels of antioxidant (supraphysiological level) resulted in reductive stress, which is as destructive to cells as oxidative stress. For instance, administration of high concentrations of ascorbate was shown to have similar effects by causing oxidative stress^85^. A balance must be attained in the use of medicinal plants with an antioxidant in order to prevent oxidative or reductive stress.

This study demonstrates that continuous and prolonged intake of aqueous extract of green tea improved sperm parameters, specifically sperm concentration and vitality, and appears to be safe in the rats. However, subtle structural changes observed in the reduced diameter and epithelial heights of the seminiferous tubule and increased spontaneous acrosome reaction needs further investigation to understand the implications on fertility outcomes.

## Methods

### Chemical reagents

All chemicals except mentioned otherwise were obtained from Sigma Aldrich (St. Louis, MO, USA).

### Preparation of aqueous extract of green tea

Green tea (*Camellia sinensis*; Five Roses™) purchased from a retail store in Cape Town, South Africa was infused in freshly boiled tap water for 5 min to final concentrations of 2% and 5% respectively, was filtered through a cheesecloth and Whatman’s filter paper (no 4 and 1, respectively) using a vacuum system (Opuwari & Monsees, 2015).

### Chemical analysis of green tea

During the period of treatment, at least three samples of prepared green tea (2% and 5%) were randomly collected at different time points and stored at − 20 °C for further use. In order to determine the quantity of soluble solids in green tea, 1 ml of 2% and 5% green tea (in triplicate) was placed in the dried vials with a known weight. It was then dried overnight at 120 °C, allowed to cool in a desiccator and weighed. The obtained results were expressed as mg/ml of tea.

Total polyphenols in green tea were determined using the Folin–Ciocalteau (F–C) reagent, with Gallic acid as standard^86^ and the result was expressed as mg Gallic acid equivalent (GAE)/ml of tea. In brief, F–C reagent (10%) and sodium bicarbonate (7.5%) was added to the tea extracts and prepared standards (5:4:1). The thoroughly mixed samples were incubated at 37 °C for 2 h and read at 765 nm.

Flavonol content was determined using quercetin as a standard, according to Mazza et al.^87^ and expressed as mg Quercetin equivalent/ml of tea. To summarise, 0.1% hydrogen chloride (HCl) in 95% ethanol (50 µl) and 2% HCl (900 µl) was added to blank, standards and diluted samples (50 µl), incubated at 37 °C for 3 min and measured at 360 nm.

Flavanol content was determined using ( +) catechin as standard^88^ and expressed as mg catechin equivalent/ml of tea. Briefly, 4-(Dimethylamino)-cinnamaldehyde (1,000 µl) was added to blank, standards and tea extract (200 µl) and incubated at 37 °C for 5 min, with absorbance read at 640 nm.

Ferric reducing antioxidant power (FRAP) was determined using Trolox as standard (0, 100, 150, 200, 400 and 600 µM)^89^, with the result expressed as mM Trolox/ml of tea. In brief, 300 µl FRAP reagent (containing 300 mM sodium acetate buffer, 10 mM 2,4,6-tripyridyl-3-triazine, and 20 mM FeCl_3_ in a ratio 10:1:1) and dH_2_0 was added to diluted tea extract (10 µl), incubated at 37 °C for 4 min and read at 593 nm using a microplate reader.

### Animals

Male Wistar rats sourced from the animal facility of the Medical Bioscience department, University of the Western Cape, had free access to standard rat chow and tap water ad libitum. The animals were kept under standard conditions in a room at a temperature of 21–24 °C with a constant 12 h light/dark cycle. The Biomedical research ethics committee of the University of the Western Cape approved this study (registration No: 11/7/40) and all experiments were performed in accordance with relevant guidelines and regulations.

### Treatment protocol

Eighteen male Wistar rats (63-day old; 200–250 g) were randomly divided into three groups (*n* = 6). Three animals per treatment group were housed in separate cages. Each group received the respective treatment ad libitum using a water bottle for 52 days:

Group one: tap water and served as control.

Group two: 2% green tea.

Group three: 5% green tea.

The amount of daily intake of fluid and the weekly bodyweight of the animals were recorded. At the end of the experiment (day 52), all rats were euthanized. Carbon dioxide was used to anaesthetize and euthanize the rats as it does not interfere with specific sperm parameters ^90^. To avoid dyspnoea and distress, rats were placed in a glass chamber which was then filled slowly with carbon dioxide from a compressed gas cylinder according to AVMA guidelines^91^. When rats lost consciousness, gas flow was increased and maintained until no respiratory movements were observed. Death was confirmed by exsanguination of the heart. Final body and organ weights were noted. Blood obtained through a cardiac puncture, allowed to clot for 30 min and centrifuged (3,000×*g*, 15 min), was stored at − 80 °C for hormonal and biochemical assays. Left cauda epididymis was excised and used for the determination of sperm concentration, vitality, motility and acrosome reaction. Testis, right epididymis, liver and kidney were fixed in Bouin’s solution for histological purposes. Left testis, liver and kidney were frozen at − 80 °C for biochemical assays.

### Serum analysis for testosterone and biochemical assays

The level of testosterone in serum obtained from the male rats was measured with an ELISA kit (EIA 1559, DRG Instruments GmBH, Marburg, Germany), as per the manufacturer’s instruction. In brief, enzyme conjugate (200 µl) was added to 25 µl of sample and standard (0, 0.2, 0.5, 1, 2, 6, and 16 ng/ml) well, mixed and incubated at room temperature (RT) for one hour. After that, contents were briskly shaken out and rinsed three times with diluted wash solution (400 µl) and stricken sharply on absorbent paper to remove residual droplet. Substrate solution (200 µl) was added to each well and incubated at RT for 15 min, and the reaction stopped by the addition of stop solution (100 µl). Absorbance was read at 450 nm, and the result expressed as nanogram per millilitre.

Serum creatinine was determined, according to Bartels et al.^92^ and expressed as mg/ml. In brief, standard solution (0, 15, 30, 60, 90, 120 and 150 mg/l creatinine in dH_2_0) or serum was added to a working solution (0.1 g picric acid in 50 ml dH_2_0 and 50 ml 0.4 NaOH in 4 °C) (1:10), incubated at RT for 15 min and measured at 492 nm.

Levels of ALT and AST in serum was determined^93^ and expressed as IU/l. ALT or AST substrate (12.5 µl) was added to serum or standard (2.5 µl) and incubated at 37 °C for 1 h. Standard solution for ALT (0, 23, 50, 83, 125 IU/l) and AST ( 0, 20, 55, 95, 145 IU/l). After that, 0.2% 2, 4-dinitrophenylhydrazine in 37% HCl (25 µl) was added to each sample and incubated for 20 min at RT. Following which, 0.4 M NaOH (250 µl) was added and incubated for a further 30 min and read at 492 nm.

Antioxidant activities in the serum were measured using FRAP as described earlier^89^ and expressed as µM Trolox/ ml of serum.

### Analysis of tissue for antioxidant activities

Previously frozen testis (20% w/v), kidney (20% w/v), and liver (20% w/v) tissues were swiftly thawed and homogenised with PRO 200 homogeniser (Proscientific Inc, Oxford, USA), in 1 ml ice-cold Tris-buffered saline (Tris–HCl 20 mM, NaCl 150 mM, pH 7.4) and centrifuged (5,000×*g*, 30 min and 4 °C). After that, the supernatants stored at − 80 °C and used for antioxidant and biochemical assays.

Lipid peroxidation was assessed according to Yagi^94^ by measuring malondialdehyde (MDA), a thiobarbituric acid reacted substances (TBARS) and represented as nanomolar of MDA per milligram of protein. In brief, thiobarbituric acid (TBA) reagent (15% v/v trichloroacetic acid and 0.25 N HCl) was added to each sample (2:1), heated at 95 °C for 15 min and allowed to cool. Samples were centrifuged (1,000×*g*; 10 min) and measured at 532 nm.

Glutathione (GSH) level was analysed as described by Ellman^95^ and expressed as millimolar thiol per milligram of protein. Briefly, Ellman’s reagent (3 ml) was added to each sample (20 µl), incubated for 15 min and absorbance read at 412 nm.

Superoxide dismutase (SOD) activity was determined by the method of pyrogallol autoxidation^96^ with a slight modification^97^. Briefly, samples were mixed with Tris-EDA-HCl buffer and pyrogallol (1:15:1) and incubated at 25 °C for 10 min. After that, 1 N HCl (50 µl) was added, and SOD activity measured at 440 nm. One unit is the amount of enzyme that inhibited the oxidation of pyrogallol by 50%, and the result represented as units per milligram of protein.

Catalase (CAT) activity was evaluated, according to Aebi^98^. In brief, samples were mixed with PBS and 20 mM H_2_0_2_ (1:4:5) and measured at 25 °C within 2 min. A unit of CAT is the activity of the enzyme that catalysed the reduction of 1 µmol of H_2_O_2_ per min per milligram of protein.

Total protein content was performed^99^ with bovine albumin serum (BSA) as standard (0, 0.2, 0.6, 1,1.4, 2.8, 4,2 and 5.6 mg/ml). To summarise, 25 µl reagent A and 200 µl reagent B (Biorad laboratory, Hercules, USA) was added to each sample and incubated at 37 °C for 30 min. Absorbance was read at 630 nm.

### Analysis of rat spermatozoa

Spermatozoa collected from freshly isolated cauda epididymis by a swim out technique was used to analyse the respective parameters^100^. Sperm diluted with phosphate-buffered saline (PBS) (1:9) was counted with Markler counting chamber (Sefi-Medical Instrument, Haifa, Israel) to determine sperm concentration. Sperm concentration was represented as millions per millilitre (10^6^/ml). Sperm vitality was determined using the fluorescent Duo vital kit (Microptic, Barcelona, Spain) according to the manufacturer’s instruction. In brief, a mixture of fluorochrome solution red (1 µl), fluorochrome solution green (1 µl) and sperm sample (5 µl), was placed on a clean slide. The slide was covered with a coverslip and spermatozoa (100) was analysed under a fluorescence microscope. Spermatozoa fluorescing green or red were respectively scored as alive or dead, and the result expressed as percentage alive. Sperm motility was determined by placing sperm suspension (5 µl) in a Leja slide (Leja Products B.V., Nieuw Vennep, Netherlands) and evaluated with Sperm Class Analyzer software (Microptic, Barcelona, Spain). Sperm parameters assessed included total motility, progressive motility, total static, curvilinear velocity (VCL, µm/s), straight-line velocity (VSL, µm/s), average path velocity (VAP, µm/s), the amplitude of lateral head displacement (ALH, µm), and linearity (LIN, %), Wobble (WOB, %), beat cross frequency (BCF, Hz) as defined^101^. Acrosome reaction was evaluated according to Larson & Miller^102^. In brief, sperm samples were fixed with 4% p-formaldehyde in PBS (1:5) for 5 min and centrifuged (3,000×*g*, 3 min). The cells were re-suspended with ammonium acetate (100 µl), smeared on a glass slide, air-dried and stained with Coomassie Brilliant Blue G (0.22%) solution for 2 min at RT. Excess dye was removed and covered with a coverslip and observed under a bright-field microscope (1000X). Spermatozoa head stained blue represented spermatozoa with intact acrosome reaction, while those with partial or no stain, spermatozoa undergoing acrosome reaction.

### Histology and morphometric measurement

Following the fixing and tissue processing of the testis, epididymis, kidney and liver, hematoxylin & eosin staining was utilised. The diameter and epithelial heights of the seminiferous tubules of the testes, and epithelial heights of the epididymis were conducted according to Opuwari & Monsees^100^. In brief, the diameters and epithelial heights of seminiferous tubules in 30 round or nearly round tubular profile per animal as well as epithelial heights of 10 caudal and caput epididymis (100X) were measured with Scope photo 3.0 (MicroImaging Ltd).

### Data analysis

Data analysis was completed with GraphPad Prism version 6.0.0 for Windows (GraphPad Software, www.graphpad.com). Kolmogorov–Smirnoff test tested for normal distribution of data. Normally distributed data were analysed with one-way analysis of variance (ANOVA) and Tukey’s multiple comparison test. Not normally distributed data were analysed with the Mann–Whitney test and Dunn’s multiple comparison test. The Independent t-test was used in the analysis of the tea extracts. ANOVA trend analysis was also employed between groups. Data were expressed as mean ± standard error of the mean, and a *P*-value less than 0.05 was set as statistically significant.
